# Research progress of T cells in cholangiocarcinoma

**DOI:** 10.3389/fimmu.2025.1453344

**Published:** 2025-02-25

**Authors:** Zhiming Wang, Yunyan Dai, Yunpeng Zhou, Yi Wang, Pinggui Chen, Yaoxuan Li, Yunfei Zhang, Xiaocui Wang, Ying Hu, Haonan Li, Gaopeng Li, Yukai Jing

**Affiliations:** ^1^ Third Hospital of Shanxi Medical University, Shanxi Bethune Hospital, Shanxi Academy of Medical Sciences, Tongji Shanxi Hospital, Taiyuan, China; ^2^ Department of Hepatobiliary Surgery, Third Hospital of Shanxi Medical University, Shanxi Bethune Hospital, Shanxi Academy of Medical Sciences, Tongji Shanxi Hospital, Taiyuan, China; ^3^ Department of Clinical Laboratory, Third Hospital of Shanxi Medical University, Shanxi Bethune Hospital, Shanxi Academy of Medical Sciences, Tongji Shanxi Hospital, Taiyuan, China

**Keywords:** cholangiocarcinoma, T lymphocytes, tumor microenvironment, immunotherapy, immunization checkpoints

## Abstract

Cholangiocarcinoma (CCA), a malignant tumor, is typically challenging to detect early and often results in a poor prognosis. In recent years, research interest has grown in the potential application of immunotherapy for CCA treatment. T cells, as a crucial component of the immune system, play a significant role in immune surveillance and therapy for cholangiocarcinoma. This article provides a review of the research advancements concerning T cells in cholangiocarcinoma patients, including their distribution, functional status, and correlation with patient prognosis within the tumor microenvironment. It further discusses the potential applications and challenges of immunotherapy strategies targeting T cells in CCA treatment and anticipates future research directions. A more profound understanding of T cells’ role in cholangiocarcinoma can guide the development of clinical treatment strategies, thereby enhancing patient survival rates and quality of life. Finally, we explored the potential risks and side effects of immunotherapy for T-cell cholangiocarcinoma.

## Graphical Abstract

Depending on the anatomical location, cholangiocarcinoma can be categorized into intrahepatic cholangiocarcinoma (iCCA), periportal cholangiocarcinoma (pCCA), and distal cholangiocarcinoma (dCCA). Research related to T-lymphocytes in cholangiocarcinoma can be carried out in depth by detecting tissues, blood and cells. By FigDraw.

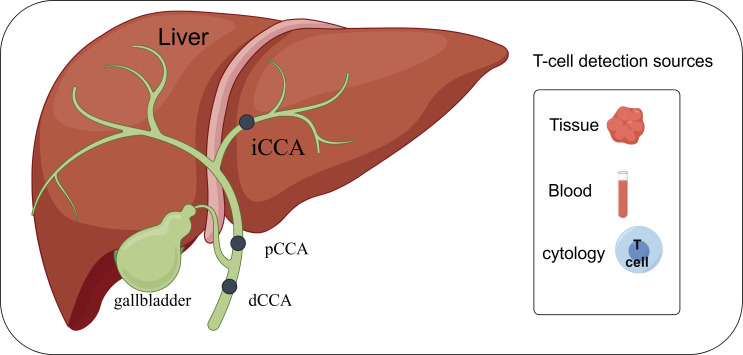

## 1 Introduction

CCA is a highly malignant neoplasm that arises from the biliary epithelium and is characterized by its late presentation and aggressive course. CCA has numerous subtypes with different origins. Intrahepatic CCA (iCCA) originates within the liver parenchyma; perihilar CCA (pCCA) occurs at the confluence of the left and right hepatic ducts; and distal CCA (dCCA) develops in the lower portion of the bile duct near the duodenum (1). Asia has the highest incidence of CCA in the world, with the proportion of CCA-related deaths ranging from 2.88% to 4.65% (2–4), posing a serious threat to public health. The etiology of CCA is multifactorial, with a range of risk factors contributing to its development. Chronic inflammation of the bile ducts, often associated with conditions such as primary sclerosing cholangitis (PSC) and chronic biliary infections, is a well-established risk factor (5). Additionally, exposure to toxins, such as certain chemicals and liver flukes, genetic predisposition, and underlying liver diseases, including cirrhosis, can heighten the risk of CCA (6). One of the greatest challenges in managing CCA lies in its insidious nature, with symptoms often remaining undetectable until the disease has advanced to later stages. Common clinical presentations include jaundice, abdominal pain, unexplained weight loss, and changes in stool or urine color (7). Recognizing these signs and symptoms early on is pivotal for timely diagnosis and intervention. Despite advancements in medical research, treatment options for CCA have been still limited to surgical intervention, chemotherapy, and radiation therapy. For patients who undergo resection, reported 5-year survival rates are low, ranging from 21 to 35% (8). Currently, the first-line chemotherapy regimen for advanced or recurrent CCA is gemcitabine plus cisplatin. However, the efficacy of chemotherapy for CCA is low compared to other cancers (9, 10). While traditional treatments like surgery, chemotherapy, and radiation have limited benefits in certain patient populations, the development of novel immunotherapeutic approaches, have begun to show potential in improving survival rates and quality of life for patients that leverages the immune system.

The effectiveness of CCA immunotherapy depends largely on the fitness and distribution of immune cells within the tumor microenvironment(TME). These factors are critical in determining which patients may benefit from such treatments. The TME of CCA consists of a diverse range of cells, including stromal cells like cancer-associated fibroblasts (CAFs), endothelial cells, and immune cells from both the innate and adaptive immune systems such as tumor-associated macrophages (TAMs), neutrophils, natural killer cells, and T and B lymphocytes (11). In CCA, T cells constitute the major subset of the TME (12). The success of some T cell-related immunotherapies developed for this purpose in cholangiocarcinoma depends on whether T cells can effectively recognize and respond to tumor antigens to attack cancer cells. Given these complexities, a comprehensive understanding of T cells’ mechanism in cholangiocarcinoma patients and related treatment strategies holds significant potential for improving patient prognosis and extending survival time. This review will encapsulate the research advancements of T cells in cholangiocarcinoma, investigate their application potential in tumor immunotherapy, and anticipate future research directions.

## 2 The basic knowledge of T cells in cancer

T lymphocytes originate from bone marrow (BM) progenitors and subsequently migrate to the thymus. After differentiating and maturing in the thymus, T lymphocytes are distributed to immune organs and tissues throughout the body, where they play a crucial role in immune responses through the circulation of lymphatic vessels, blood, and tissue fluid (13). Over the past few decades, we have been studying T cells more and more, and our knowledge of T cells has become clearer and clearer. In this section, we describe several of the major T cell subsets to aid in the understanding of this review.

CD4^+^ T helper (Th) cells represent a heterogeneous group of T cells that play central roles in almost all aspects of immune responses. These cells can be activated by the peptide-MHC class II complex on antigen-presenting cells (APCs), along with costimulatory signals and cytokine signaling, differentiating into several subsets characterized by distinct surface molecules and cytokine profiles, including Th1, Th2, Treg, Th17, etc (14, 15).

Th1 cells predominantly exert anti-tumor activity. The frequency of the Th1 subset and the production of IFN-γ in the TME correlate positively with better clinical outcomes across multiple tumor types including melanoma, breast, ovarian, lung, colorectal, and laryngeal cancers (16–22). Th1 cells promote tumor rejection by shaping an anti-tumor immune environment and indirectly supporting the effector functions of other immune cells (23). They are an important subset of CD4 T cells that provide help for CD8 T cell responses and functions. The migration of effector CD8 T cells in the TME depends on the chemokine receptor CXCR3 and its ligands, CXCL9 and CXCL10, which are predominantly expressed by Th1-related, IFN-γ-activated macrophages, cancer-associated fibroblasts (CAFs), and tumor cells (24). Additionally, IFN-γ and IL-2 produced by Th1 cells enhance the survival, proliferation, and cytolytic function of CD8 cytotoxic T lymphocytes (CTLs) (25). IFN-γ can significantly enhance MHC class I and II expression, as well as tumor-derived antigen presentation on tumor cells (26).

The role of Th2 cells in tumor progression remains controversial, exhibiting both favorable and deleterious effects. Previous studies have shown that Th2 cells can suppress tumor growth by activating eosinophils as cytotoxic effector cells in murine plasmacytoma and melanoma (27). The adoptive transfer of tumor-specific Th2 cells induces a massive accumulation of M2-type macrophages at the tumor site, triggering an inflammatory immune response to eliminate myeloma cells (28). However, Th2-associated IL-4 signaling in monocytes and macrophages promotes breast cancer metastasis (29). Th2 cells can also attenuate Th1-associated anti-tumor responses through IL-4 signaling (30). The discrepancies in Th2-mediated tumor immunity may be attributed to different tumor types and distinct Th2 cell states. For example, studies suggest that tumor-promoting Th2 cells exhibit high levels of IL-10 and TGF-β, whereas Th2 cells with elevated expression of IL-3, IL-5, and IL-13 demonstrate anti-tumor immunity (31, 32). Regulatory T (Treg) cells are a specialized subset of CD4 T cells that maintain immune tolerance by suppressing immune responses. Treg cells are characterized by high expression of the IL-2 receptor alpha chain (IL-2Rα, CD25), inhibitory cytokines IL-10, TGF-β, and IL-35, as well as the master transcription factor Foxp3 (33). Two major subsets of Treg cells are identified based on their developmental origin: thymic Treg (tTreg) cells, also known as natural Treg (nTreg) cells that derive from the thymus, and induced Treg (iTreg) cells that differentiate from conventional CD4 T cells in the periphery following antigen stimulation in the presence of TGF-β and IL-2 (34). Treg cells are significantly infiltrated in many solid tumors (35, 36), and a high frequency of Treg cells is mainly associated with worse clinical outcomes in the majority of tumor types.

**Figure 1:**
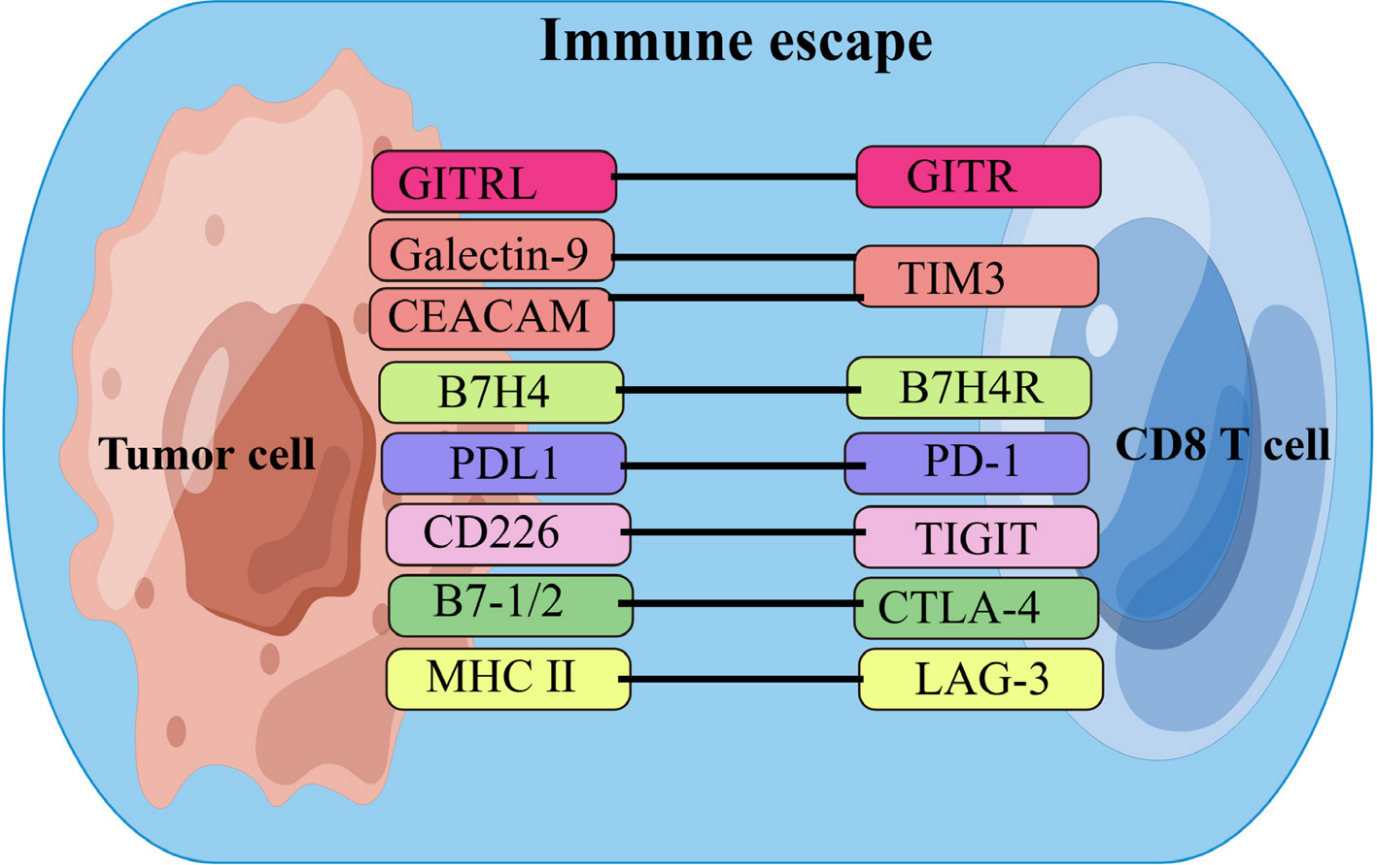
List of ICs and their receptors in CCA. Cholangiocarcinoma cells achieve immune escape by interacting with immune checkpoints on CD8^+^T cells. ICs, immune checkpoints;B7H4, B7 homolog 4; B7H4R, B7H4 receptor; PD1, programmed cell death protein-1;PDL1, programmed cell death ligand-1; CEACAM, the carcinoembryonic antigen-related adhesion molecules; MHC II, major histocompatibility complex class II; GITR, Glucocorticoid-Induced TNF-related protein; GITRL, GITR ligand; TIM3, T cell immunoglobulin and mucin domain-containing protein 3;CTLA-4, cytotoxic T-lymphocyte associated protein 4; TIGIT, T-cell immunoglobulin and ITIM domain; LAG-3, lymphocyte activation gene 3. By FigDraw.

CD8^+^ T cells play critical roles in combating intracellular pathogens and eliminating malignant cells in cancer (37). Upon antigen stimulation, naïve CD8^+^ T cells undergo robust expansion, giving rise to effector and memory T cells. Effector CD8^+^ T cells, known as CD8^+^ cytotoxic T lymphocytes (CTLs), can directly induce target cell death through the interaction between Fas and its ligand, as well as the secretion of the cytolytic mediator perforin, which creates pores in target cells and allows the delivery of granule serine proteases (granzymes) to induce apoptosis. Memory CD8^+^ T cells provide rapid and strong protection upon antigen re-encounter, which is critical for effective and long-term immunity. During CD8^+^ T cell differentiation, heterogeneous effector and memory populations have been identified, including short-lived effector CD8^+^ T cells (TE), exhausted CD8^+^ T cells (Tex), long-lived memory CD8^+^ T cells (TM), memory precursor CD8^+^ T cells (TMP), central memory CD8^+^ T cells (TCM), effector memory CD8^+^ T cells (TEM), and tissue-resident memory (TRM) cells, named for their phenotype, differentiation potential, and functionality (38, 39). With tumor progression, CD8^+^ T cells gradually lose their production of IL-2 and TNF-α, as well as their cytotoxic function (40). A key hallmark of Tex cells is the upregulated and sustained expression of multiple immune checkpoints(ICs), such as PD-1, CTLA-4, TIGIT, Tim-3, LAG-3, and GITR. Tumors undergo immune escape via these immune checkpoints by destroying CD8^+^ T cells or inhibiting their immune function, thus achieving tumor immune escape for tumor metastasis or progression (41–45). **Figure 1** shows the currently known CD8^+^ T cell-related immune checkpoints and their receptors in CCA. The extent and co-expression of ICs directly correlate with the severity of exhaustion (46). On the other hand, Tex cells also express costimulatory molecules, which can promote T cell exhaustion in the tumor microenvironment. For example, costimulation of CD27 and CD28 enhances T cell exhaustion (47). CD28 signaling is compromised due to loss of competition with CTLA-4 for B7 family ligands (48). PD-1 signaling further suppresses T cell function by specifically inducing CD28 dephosphorylation (49).

Tissue-resident CD8^+^ T cells, identified as CD103^+^ CD8^+^ T cells, are essential for the anti-tumor immune response in regional tissue immunity (50). E-cadherin is an important ligand for CD103 (integrin alpha E, ITGAE) (51). In the tumor microenvironment (TME), epithelial cancer cells can express E-cadherin, interact with CD103^+^ CD8^+^ T cells, and maintain the interaction with cancer cells, leading to the residence of tumor antigen-reactive CD8^+^ T cells and a persistent anti-tumor effect in tumor tissues (52). It was found that patients with high infiltration of tissue-resident CD8^+^ T cells in ICC tumor tissues had better overall survival (OS) and prognosis (53).

## 3 The proportion and distribution of T cells in cholangiocarcinoma

Studies have demonstrated that the proportion and distribution of T cell subsets significantly alter in patients with cholangiocarcinoma. Specifically, the study discovered an increase in exhausted and regulatory T cells, a reduction in cytotoxic T cells, and the appearance of tumor-specific T cells in cholangiocarcinoma tissue (54, 55). The proportion of total lymphocytes decreased, while the percentages of activated T cells as well as CD4^+^CD25^+^ regulatory T cells (Tregs) increased in peripheral blood of patients with CCA (56). Additionally, tumor-infiltrating immune cells were found to be concentrated in the tumor stroma and infiltration margins, yet scarce in the tumor epithelium and tumor core. Overall, CD8 T cells showed high expression of suppressive markers (PD-1, TIM-3, LAG-3, TIGIT, and NKG2A), indicating depletion of cytotoxic effector cells, along with high infiltration of immune-suppressing tumor-infiltrating Tregs (CD4FOXP3) (57), and also the impaired function of tumor-specific CD8^+^ T cells and enhanced immunosuppression by CD4^+^ regulatory T cells (58).Regarding the spatial distribution of T cell subsets, the study discovered that the density of CD8^+^ T cells, FoxP3^-^CD4^+^ helper T cells, and FoxP3^+^ CD4^+^ regulatory T cells in the tumor edge area was considerably higher than that in the tumor stroma and tumor core (59). Additionally, the density of tissue-resident CD8^+^ tumor-infiltrating lymphocytes (TILs) expressing CD69^+^CD103^+^ was noticeably higher in the tumor edge zone and tumor core zone than in the stromal zone (53, 60). Spatial heterogeneity is one of the key features of the tumor microenvironment (61), and the composition and localization of the immune infiltrate varies significantly according to its dynamic interactions with tumor and/or stromal cells (62, 63). According to the above studies, the peritumor region rather than the tumor core itself is the main site of active infiltration of T cell subsets such as CD8^+^ T cells and FoxP3^-^CD4^+^ T cells, while Tregs infiltrate into the tumor. Thus, CCA must be considered an immune-rejecting tumor in which the majority of effector T cells are isolated at the tumor margin (64).These results highlight the complex changes of T cells in the cholangiocarcinoma microenvironment and provide potential immunotherapy directions, providing an important reference for optimizing immunotherapy strategies for cholangiocarcinoma. An overview of the different cell subsets and their spatial distribution is presented in **Table 1**.

**Table 1:** Characteristic distribution of T lymphocytes in CCA.

Ref	Year	Country	Location of TILs	Experimental materials	Experimental methods and assessment of TILS	Outcomes
(54)	2023	China	IT vs PT	ICC tissue	ScRNA and scTCR sequencing	1.exhausted and regulatory T cells:IT>PT 2.cytotoxic T cells:IT<PT 3.IT: new tumor-specific T cell
(57)	2023	China	IT vs TM	ICC tissue	multiplexed immunofluorescence	tumor-infiltrating immune cells:TM>IT
(58)	2022	Italy	IT vs TM	ICC tissue	high-dimensional single-cell technologies	1.tumor-specific CD39^+^ CD8^+^ T cells:TM>IT 2.hyperactivated CD4^+^ Tregs: TM>IT
(59)	2021	Korea	IT vs TM	CCA tissue	mIHC	1.FoxP3^-^ CD4^+^ helper T cells, FoxP3^+^ CD4^+^ regulatory T cells and CD8^+^ T cells: TM>IT 2.FoxP3^-^CD4^+^ helper T cell and LAG3^+^TIM3^+^CD8^+^ T cell: TM>IT
(53)	2023	China	IT vs PT	ICC tissue	mIHC	Tissue-resident CD8T cells (CD103CD8T cells): IT>PT
(60)	2021	Korea	Blood vs ICC tissue	Blood ICC tissue	multicolor flow cytometry mIHC RNA sequencing	CD69^+^CD103^-^and CD69^+^CD103^+^tissue-resident memory (TRM) -like CD8^+^T cells: Blood < ICC tissue

## 4 Molecular pathogenesis of T cell-associated cholangiocarcinoma

The molecular pathogenesis of T cell-associated cholangiocarcinoma encompasses several facets. Chronic inflammation is a major contributor to cancer promotion and progression. A plethora of clinical and epidemiological observations have validated the link between a prolonged inflammatory state and cancer incidence. Primary sclerosing cholangitis (PSC) and primary biliary cholangitis (PBC) are major chronic inflammatory diseases that damage bile duct epithelial cells. Although PSC predisposes individuals to bile duct cancer, its incidence is low in the autoimmune setting of PBC. Type 1 T helper (Th1) and T cytotoxic (Tc1) effector cells are critical mediators of both autoimmunity and cancer immunosurveillance (65). In PBC mice, Th1/Tc1 and Th2/Tc2 cell subsets were notably enriched in the liver, detected in tumor-draining lymph nodes, and concentrated in CCA tissues compared with PSC mice or mice without cholangitis (66). This suggests that protection against PBC depends on both type 1 and type 2 T cell responses.

Cholangiocarcinoma cells evade immune surveillance by obstructing Fas receptor (FasR) signaling or augmenting Fas ligand (FasL) expression to trigger apoptosis in T cells. Further research revealed that decreasing the expression of FLICE inhibitory protein (I-FLICE) in cholangiocarcinoma cells reinstates Fas-mediated cell apoptosis. I-FLICE is homologous to cystatinase 8 and expresses a death effector structural domain but has no catalytic activity. Therefore, it competitively prevents the binding of cystatinase 8 to the FasR complex by binding to FADD (Fas-associated with death domain protein) through its death effector domain, thus preventing Fas-mediated apoptosis. Hence, suppressing the expression of I-FLICE could prove beneficial for cholangiocarcinoma treatment (67–69).

In addition, studies have discovered that cholangiocarcinoma cells overproduce mucin 1 (MUC1), which interacts with EGFR, thereby activating the EGFR/PI3K/Akt signaling pathway. Simultaneously, this interaction provokes the accumulation of Foxp3^+^ regulatory T cells in the tumor microenvironment, enhancing the malignant phenotype of cholangiocarcinoma cells and promoting tumor initiation. Consequently, this process intensifies the growth and metastasis of cholangiocarcinoma (70). However, it is unclear how MUC1 regulates the enrichment of Foxp3^+^ Treg cells in the TME. Many questions, including which cytokines are involved, how Foxp3^+^ Treg cells respond to these induced signals, and the source of Foxp3^+^ Treg cells, need to be further explored.

Furthermore, the expression of MMP14 in cholangiocarcinoma tissue is significantly elevated compared to adjacent tissues. MMPs (matrix metalloproteinases) are a group of proteinases intimately linked with angiogenesis and tumor progression (71).MMP14, the first transmembrane protein identified in this group, is strongly correlated with the infiltration of various immune cells. The number of central memory CD8 T cells, neutrophils, monocytes, and central memory CD4 T cells was significantly decreased in patients with ICC with high MMP14 expression. MMP14 may accelerate the progression of ICC by interfering with the abundance of monocytes and CD4 T cells (72).

Mutations in isocitrate dehydrogenase 1 (mIDH1) are prevalent in cholangiocarcinoma (73–75). The mIDH1 enzyme produces (R)-2-hydroxyglutarate, which in turn supports the maintenance of cholangiocarcinoma tumors with an immune evasion program centered on a dual mechanism mediated by (R)-2-hydroxyglutarate (suppression of CD8^+^ T cell activity and tumor cell-autonomous inactivation of TET2 DNA demethylase) (76, 77).

Lastly, a substantial upregulation of secreted phosphoprotein 1 (SPP1) has been observed in the tumor epithelial cells of ICC. CD44 was identified as a ligand for osteopontin (OPN), a protein encoded by SPP1, which is primarily expressed in T cells. SPP1 is thought to inhibit T cell activation, however, how the SPP1-CD44 combination affects T cell anti-tumor immunity and clinical outcomes in patients remains unclear (78). SPP1 interacts with T cells via SPP1-CD44 interaction, inhibiting the sustained proliferation of T cells. However, immunosuppressive T cells in the TME may evade this inhibition by reducing CD44 expression (79). Collectively, these research findings uncover the molecular mechanisms intimately linked with T cells and cholangiocarcinoma pathogenesis, offering vital insights for the formulation of new immunotherapy strategies and prognostic markers. **Table 2** provides a summary of the molecular pathogenesis of CCA associated with T lymphocytes collected for this review.

**Table 2:** Molecular pathogenesis of CCA associated with T lymphocytes.

Ref	Year	Country	Study of genes or molecular pathways	Experimental materials	Experimental methods and assessment of TILS	Tumor type	Main findings
(67)	2017	Italy	Fas/FasL pathway	cell	cell culture, Western blot, IHC	iCC	iCCA cells have immunomodulatory properties and mediate T cell apoptosis through the Fas/FasL pathway.
(70)	2023	China	EGFR/PI3K/Akt signaling pathway	Cell tissue	cell culture, Western blot, IHC	CCA	MUC1 interacts with EGFR and activates the EGFR/PI3K/Akt signaling pathway, thereby inducing the aggregation of Foxp3^+^Treg cells, enhancing the malignant phenotype of cholangiocarcinoma cells, and ultimately promoting the growth and metastasis of cholangiocarcinoma.
(72)	2023	China	MMP14	tissue	Sangshin database analysis	iCC	MMP14 affects the infiltration of activated-memory CD4^+^T cells, resting-memory CD4^+^T cells, and other immune cells, and is strongly associated with the expression of CD200, CTLA-4, CD14, CD44, and other immune checkpoints.
(79)	2023	China	SPP1	tissue	Sangshin database analysis	iCC	SPP1 expression is upregulated in ICCA tumor epithelial cells, and SPP1-CD44 interactions impede T cell proliferation, but immunosuppressive T cells in the TME may escape this suppression by reducing CD44 expression.
(76)	2022	America	IDH1	tissue	RNA-seq IHC,HE,ELISA	iCC	Mutant-IDH1 inhibits cytotoxic T cell function through the IFNγ-TET2 axis

## 5 T cells play a pivotal role in the prognosis of cholangiocarcinoma

In CCA patients, the extent of CD8 T cell infiltration in tumor tissues exhibits a negative correlation with serum alpha fetoprotein (AFP)levels, tumor size, and lymph node metastasis (80). Low-level CD8 T-cell infiltration corresponds to shortened OS and shortened disease-free survival (DFS) (80–83).Among patients with ICC, those with a higher ratio of CD8^+^ PD-1^High^ in CD8^+^ PD-1^+^ cells experience poorer postoperative survival (84).This might be due to the expression of PD-1^High^ suggesting highly activated CD8^+^ T cells, which, however, demonstrate severe functional dysregulation and impaired IFN-γ secretion, leading to negative clinical outcomes (85). A high proportion of CD8^+^ PD-1^High^ in activated CD8^+^ PD-1^+^ cells leads to CD8^+^ T cell exhaustion (84). Research also indicates that late recurrence patients with ICC have higher levels of regulatory T cell infiltration in the TME and lower CD8^+^ T cell infiltration compared to early recurrence patients (86).Moreover, the expression levels of T cell chemokines, such as CXCL9, CXCL10, and CXCL11, are lower in the TME of late recurrence patients (86).

In ICC patients, the FoxP3 to CD8 tumor-infiltrating lymphocytes ratio (FCR) is linked with poor prognosis and lymph node metastasis (87).ICC patients with a higher FCR show poorer recurrence-free survival and OS, and those with lymph node metastasis have a higher FCR in tumor-free lymph nodes (TFLN) compared to patients without lymph node metastasis (87).FoxP3^+^ Treg cells can be categorized into three subtypes: Treg I (CD45RA^+^FoxP3^low^), Treg II (CD45RA^−^FOXP3^high^), and Treg III (CD45RA^−^FoxP3^low^) (88, 89).The Treg III subtype within regulatory T cells (Tregs) may significantly influence the prognosis of ICC patients. Studies have found that the Treg III subtype is predominant in the peripheral blood and tumor tissues of ICC patients and is associated with higher rates of recurrence-free survival. However, Treg I and Treg II are not associated with ICC recurrence (86). In previous studies, FoxP3^+^ Treg cells have always been reported to be associated with poor outcomes in cancer patients. However, there are some opposite findings in hepatocellular carcinoma and vulvar melanoma (90, 91). The roles of FoxP3 expression levels and FoxP3^+^ Treg cells in predicting prognosis of biliary malignancies have rarely been investigated, and thus need to be abundantly confirmed by more studies. Further, analysis of peripheral blood mononuclear cells from CCA patients and healthy volunteers revealed that lower levels of helper T cells (HT), higher levels of effector regulatory T cells (eTregs), and lower levels of CD80^+^ eTregs are associated with shorter overall survival. Recurrence in CCA patients is associated with higher frequencies of CD4^+^ T cells, CCR6^+^ nTregs, and CXCR3^+^ nTregs, and lower frequencies of PD-1^+^ HT, OX40^+^ HT,CD8^+^ T cells, and CTLA-4^+^ CD8^+^ T cells (92).

Mucosal associated invariant T (MAIT) are cytotoxic innate T cells that are highly enriched in the human liver near the biliary epithelium, and are reduced in tumors of patients with intrahepatic and perihepatic CCA. The researchers found that patients who retained large numbers of MAIT cells in their tumors and surrounding liver tissue had a higher likelihood of long-term survival (93).In conclusion, T cells are intimately linked with the prognosis of cholangiocarcinoma patients. Predictions about patient outcomes can be made based on their functional status, infiltration level, and subtype distribution. **Table 3** shows the relationship between T-lymphocytes and CCA prognosis collected for this review.

**Table 3:** Relationship between T-lymphocyte CCA prognosis.

Ref	Year	Country	Tumor type	Assessment Of TIL	Follow-up (months)	Endpoint	Prognostic significance
(80)	2021	China	ICC(140)	IHC	25(median)	OS/RFS	Patients with high CD73 expression in ICC tissue or too few tumor-infiltrating CD8^+^ T cells exhibit shorter OS and higher DFS
(81)	2018	Japan	ECC(114)	IHC	62.6(median)	OS	Low CD8^+^ T cells and high Foxp3^+^ Treg cell infiltration in ECCA tumor tissue are associated with poorer OS
(82)	2022	China	CCA(104)	mIHC	>36	OS	1. High Treg cells in CCA tissues are significantly associated with poor prognosis 2. High granzyme-BCD8 effector T cells in ICC and dCCA tissues were significantly associated with better OS
(84)	2020	China	ICC(322)	mIHC	27 (median)	OS/TTR	Higher proportion of CD8^+^ PD-1^High^ in CD8^+^ PD-1^+^ cells had poorer OS
(86)	2023	China	ICC(99)	mIHC	Not reported	RFS	1. Patients with high FoxP3^+^ Treg cell infiltration in ICC tumor tissues have longer RFS, which is an independent favorable prognostic factor. 2. TregIII in peripheral blood correlates with RFS in patients with ICC.
(87)	2022	Japan	ICC(61)	IHC	27.5(median)	OS	Intratumoral FoxP3^+^ Treg is associated with CD8^+^ T-cell infiltration, and a high FoxP3/CD8 ratio (FCR) is an important marker of poor survival
(92)	2021	Japan	CCA(41)	flow cytometry(peripheral blood)	20(median)	OS/RFS	Low infiltration of helper T cells (HT), high infiltration of effector regulatory T cells (eTregs), and low infiltration of CD80^+^ eTregs were associated with shorter OS
(93)	2022	Sweden	ICC	IHC	20(median)	OS	High tumor infiltration of MAIT cells is associated with good immune adaptation and predicts long-term survival in CCA patients

## 6 Immunotherapeutic approaches for T-cell-associated CCA

### 6.1 ICIs

Immune checkpoint inhibitors(ICIs) are monoclonal antibodies that focus primarily on immune checkpoint regulatory molecules. CTLA-4 and PD-1 represent the most classical T-cell immune checkpoints and are the most widely studied targets for ICIs (94). PD-1 is a typical representative with intrinsic and extrinsic mechanisms of induction, in which the extrinsic mechanism, also known as adaptive resistance, refers to the adaptation of PD-L1-expressing tumors to antitumor immunity (95). PD-L1 primarily limits the ability of T cells to mount an immunological defense by attaching to PD-1. The binding of PD-L1 to PD-1 on T cells is how this occurs. Depletion of T cells results from this process; however, PD-L1/PD-1 inhibition can also be used to restart the antitumor response (96). CTLA-4 is particularly aberrantly strongly expressed in Tregs and is frequently expressed on activated CD4^+^CD8^+^ T cells. In order to prevent T cell activation, active T cells produce CTLA-4, a CD28 homolog, which competes with CD80/86 for binding to CD28. Blocking the CTLA-4 signaling pathway significantly improves the immune response and lessens T cells’ tendency to become suppressive.

**Figure 2:**
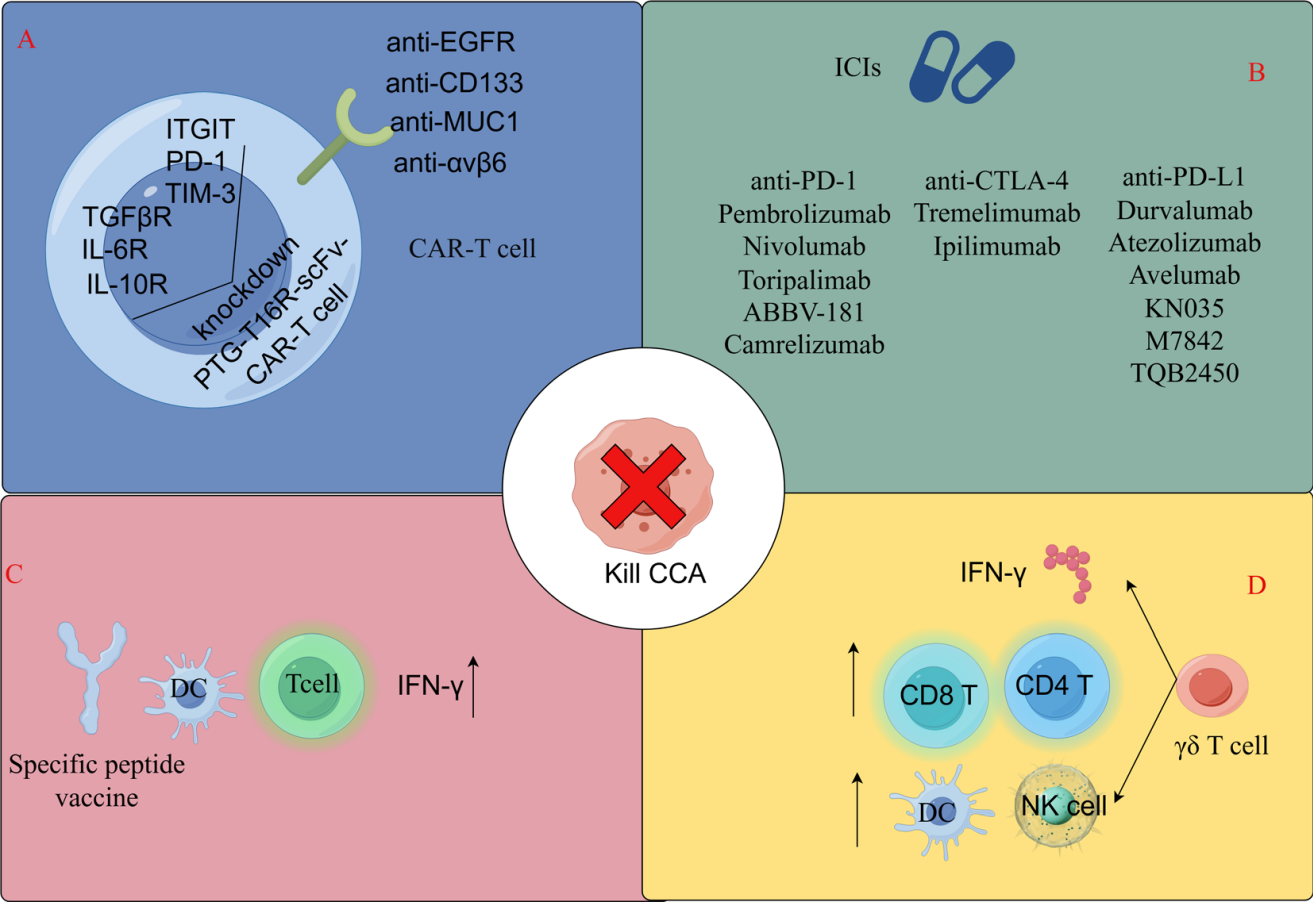
Immunotherapeutic approaches for T-lymphocyte-associated CCA. **(A)** CAR-T cell therapy. **(B)** Immune checkpoint inhibitors for cholangiocarcinoma in clinical studies or early stage trials. **(C)** Specific peptide vaccination therapy. **(D)** AllogeneicγδT cell immunotherapy. By FigDraw.

Gemcitabine treatment with CCA cells upregulates the expression of an immune checkpoint protein (PD-L1), thereby inhibiting the cytotoxicity of T lymphocytes. To overcome this challenge and take advantage of PD-L1 upregulation after gemcitabine treatment, investigators produced a recombinant PD-L1xCD3 bispecific T-cell attractor that specifically binds to CD3 on T-lymphocytes as well as PD-L1 overexpressed on CCA cells after gemcitabine treatment, thereby simultaneously blocking PD-1/PD-L1 signaling and recruiting T-lymphocytes to eliminate CCA cells. The results showed that the cytotoxicity of T lymphocytes against CCA cells was significantly enhanced, especially after gemcitabine treatment, and the cytotoxicity was positively correlated with the level of PD-L1 expression. The combination of gemcitabine and PD-L1xCD3 conjugate has been shown to be a potential alternative therapy for the treatment of CCA (97) (**Figure 2B**).

### 6.2 Advanced cell therapy

Advanced cell therapy (ACT), a type of cancer immunotherapy that uses a patient’s own immune cells to locate and destroy tumor cells, was developed as a result of advancements in solid cancer research and technological discoveries. Its main objective is to destroy cancer cells by altering or triggering the immune system of patients. Tumor-infiltrating T lymphocytes are considered involved in ACT, among other processes. Tumor-infiltrating lymphocytes(TILs) are specifically taken from surgically removed tumor samples, activated and grown in a laboratory, and then returned to the patient. TIL ACT, as a therapeutic agent, has been shown to have objective anticancer effects in numerous solid cancers (98–100), including CCA (98). Chimeric antigen receptor-T (CAR-T) cell therapy is becoming increasingly well known as a cutting-edge method for treating cancer (101). Immunotherapy using chimeric antigen receptor-modified T cells (CARTs) is a unique approach for treating a variety of malignant tumors. Choosing the right antigen on cancer cells is crucial for designing a CAR-T-cell strategy that works and avoids side effects.

**Figure 3:**
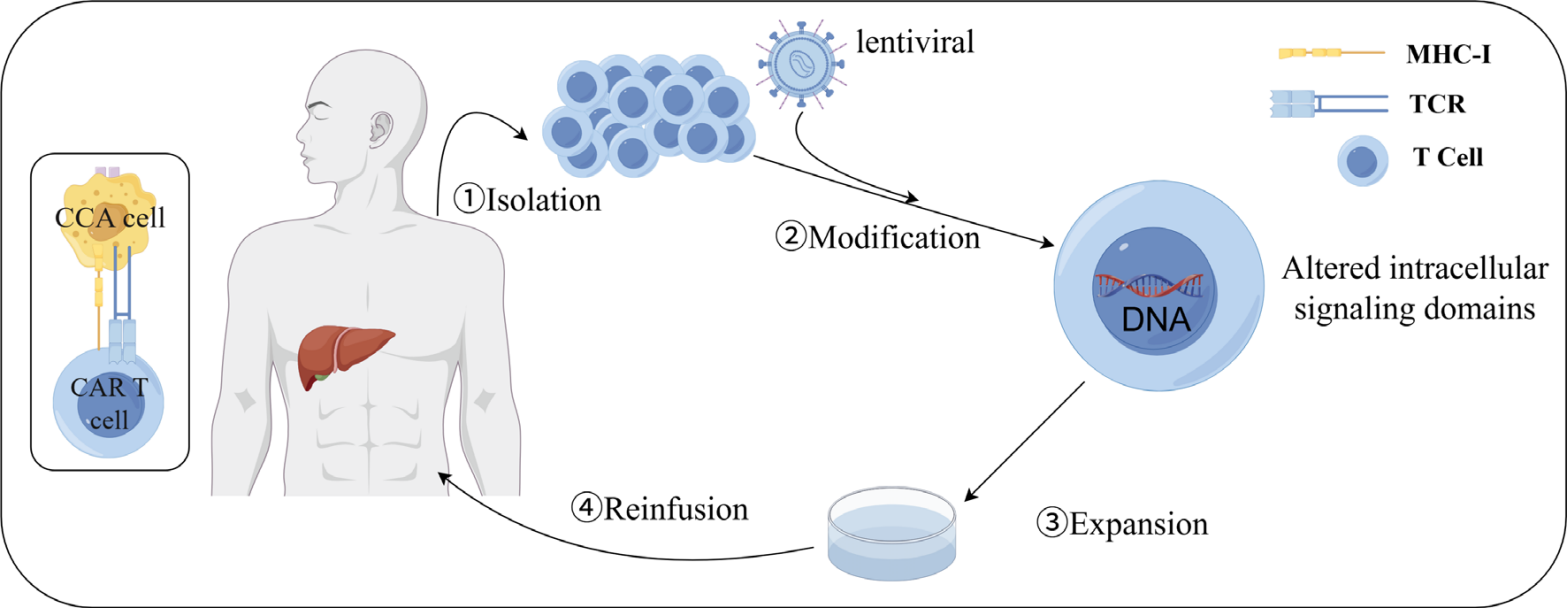
Preparation of CAR-T cells. **(1)** isolation: PBMCs were collected from peripheral blood of patients or donors; **(2)** modification: T cells were activated and CAR was transduced into activated T cells by lentivirus; **(3)** expansion: modified T cells were expanded *in vitro* to obtain clinically relevant cell counts; **(4)** reinfusion: modified T cells at the desired dosage were reinfused into patients who were previously lymphocyte-depleted. By FigDraw.

CAR-T cells (**Figure 3**), specifically targeting the epidermal growth factor receptor, can be employed in the treatment of advanced cholangiocarcinoma cases (102).CD133, a recognized cancer stem cell marker, is highly expressed and linked with cancer progression. Anti-CD133-CAR4 T cells demonstrate high potency against CD133-expressing CCA cells, leading to tumor cell lysis in a dose- and CD133 antigen-dependent manner (102, 103). MUC1, an overexpressed protein in CCA cells, is a potential target antigen for CART cell therapy. Integrin αvβ6 is upregulated in CCA but expressed at a low level in normal epithelial cells (104, 105), suggesting that integrin αvβ6 is an attractive target antigen for CAR T cell immunotherapy in CCA. Research has found that CAR-T cells targeting integrin αvβ6 and mucin 1, expressed on bile duct cancer cells, can be utilized in adoptive T cell therapies for bile duct cancer (106–109).However, MUC1 overexpression is also linked with the upregulation of PD-L1, an immune checkpoint protein that inhibits the antitumor function of T cells, which may lead to reduced efficacy of MUC1-targeting CART cell therapy for cholangiocarcinoma. To address this, researchers developed an anti-MUC1-CART cell line, αM.CAR/SRT, which contains a PD-1-CD28 switch receptor (SR) that targets MUC1 and engages the inhibitory PD-1/PD-L1 interaction to trigger CD28 signaling. Compared to αM.CAR cells, the αM.CAR/SRT cells display augmented cytotoxic function against CCA cells (110). Three immune checkpoints with the highest expression of PD-1, Tigit and Tim-3, as well as three key soluble immunosuppressive cytokines, TGFβR, IL-10R and IL-6R, were screened from cholangiocarcinoma tissues. PTG-T16R-scVF-CAR-T cells were designed based on these six tumor immunosuppressive targets, and both *in vivo* and *in vitro* experiments showed that this T-cell therapy has a strong inhibitory effect on CCA tumor growth (111) (**Figure 2A**).

### 6.3 Specific peptide vaccination therapy

Dendritic cells (DCs) are antigen-presenting cells that take up antigens and present them to adaptive immune cells. CCA tumor tissues have a higher population of activated DCs compared to normal tissues, suggesting that DCs are involved in CCA (112). DCs play an important role in enhancing antitumor responses, and the absence of DCs or the presence of dysfunctional DCs can lead to adverse outcomes. Therefore, increasing DC density and/or restoring DC function can be considered as a potential therapeutic approach for treating malignancies including CCA. Vaccines targeting DCs are another strategy to promote antitumor immunity (113). DC vaccines are usually pulsed with tumor-associated antigens (TAAs) *in vitro* and then injected *in vivo*. Several TAAs have been studied in CCA (114–116). The researchers chose three cholangiocarcinoma driver mutations (TP53, KRAS, and RNF43) to design antigenic peptides and used the antigenic peptides to stimulate DCs during DC differentiation. Peptide treatments had no effect on the differentiation of DCs to monocytes but increased the gene expression levels of the CD80 and CD86 costimulatory molecules, which play a role in regulating the interactions between DCs and T cells and in activating T cell function. Increases in CD80 and CD86 following peptide stimulation enhance T cell-DC interactions and function. DC-activated T cells stimulated by antigenic peptides had higher populations of IFN-γ-positive CD4^+^ and CD8^+^ cells, which enhanced the killing ability of cholangiocarcinoma cells (117) (**Figure 2C**).

### 6.4 γδ T cells

A major immune evasion strategy found in advanced cancers is the downregulation of MHC molecules that are required for αβ T cell activation upon presentation of somatically mutated “neoantigens” to the αβ T cell receptor (118). However, this limitation does not apply to T cells expressing γδ TCR (γδ T cells), which, although rare in human peripheral blood, are enriched in epithelial tissues where many cancers develop and have been shown to actively participate in antitumor immunity (119). γδ T cells make up a small fraction, ranging from 1% to 10%, of the total human CD3^+^ T-cell population. These cells express a lineage-specific TCR, containing one of seven Vγ chain isotypes (Vγ2, 3, 4, 5, 8, 9, and 11) paired with one of four Vδ chain types (Vδ1, 2, 3, and 5), which can be highly diverse due to the stochastic nature of the TCR somatic recombination process (120). Researchers sorted and cultured Vγ9Vδ2T cells from healthy human peripheral blood PBMCs and co-cultured them with cholangiocarcinoma cell lines, and showed that Vγ9Vδ2T cells could mediate cholangiocarcinoma apoptosis via lysosome-associated membrane protein (LAMP-1), suggesting that Vγ9Vδ2T cells may be useful in facilitating the development of new strategies for adoptive immunotherapy of cholangiocarcinoma (121, 122). This is related to the fact that γδ T cells recognize antigen in a non-MHC-restricted manner and that γδ T cells provide an early source of IFN-γ in the tumor microenvironment, γδ T cells can enhance the function of CD4^+^, CD8^+^ T cells, mature dendritic cells and activate neutrophils (123–125). Among human γδ T cell subsets, Vδ2^+^ T cells (especially those expressing Vγ9Vδ2 TCR) have been more extensively studied because they are the most abundant subset in peripheral blood. However, in skin cancer tissue infiltration, the number of Vδ1 T cells exceeds that of Vδ2 T cells. Compared with Vδ2 TIL cultures, Vδ1 tumor-infiltrating lymphocyte (TIL)-derived cell cultures can exhibit superior *in vitro* cancer killing ability (126, 127), and Vδ1 T cells can exist as tumor-reactive lymphocytes for a long time (128), so the study of Vδ1 T cells in the treatment of cholangiocarcinoma needs to be carried out (**Figure 2D**). **Table 4** and **Figure 2** demonstrate the immunotherapeutic approach to T-lymphocyte-associated CCA.

**Table 4:** Potential T-lymphocyte-associated immunotherapies for CCA.

Ref	Year	Country	Experimental methods	Tumor type	Treatment	Outcomes
(102)	2017	China	clinical trial	CCA	CART cells targeting EGFR and CD133	This patient achieved longer progression-free survival with CART-EGFR and CART133 therapy.
(103)	2020	Thailand	Cell culture experiment	CCA	CART cells targeting CD133	Anti-CD133-CAR4T Cell Immunotherapy available to treat patients with CD133-Positive CCA tumor cells
(106)	2021	Thailand	Cell culture experiment	CCA	CART cells targeting MUC1	Anti-MUC1-CAR4 T cells achieve anticancer effects on MUC1-expressing CCA cells by increasing the production of anti-tumor cytokines (TNF-α and IFN-γ), pro-apoptotic proteins (granzyme B), and by inducing lysis of CCA cells.
(107)	2021	Thailand	Cell culture experiment	CCA	CART cells targeting integrin αvβ6	Anti-αvβ6-CAR T cells effectively kill αvβ6-positive CCA cells
(108)	2023	China	Cell culture experiment	ICC	CART cells targeting Tn-MUC1(5E5)	Anti-5E5-CAR T cells effectively eliminate Tn-MUC1-positive ICC cells *in vitro* and *in vivo*
(110)	2023	Thailand	Cell culture experiment	CCA	aM.CAR/SR T cells: anti-MUC1-CAR (aM.CAR) T cells containing SR molecules (PD-1-CD28)	aM.CAR/SR T cells were significantly more cytotoxic to CCA cells expressing MUC1 and PD-L1
(111)	2023	China	Cell culture experiment	CCA	PTG-T16R-scFv-CAR-T cells	PTG-T16R-scFV-CAR-T cells knocking down the hexameric inhibitory molecule are highly immune to cholangiocarcinoma cells *in vivo* and *in vitro*
(97)	2022	Thailand	Cell culture experiment	CCA	Combination gemcitabine and PD-L1xCD3 bispecific T cell engager (BiTE)	BiTE significantly enhanced the cytotoxicity of T lymphocytes against CCA cells, especially after gemcitabine treatment, and the magnitude of cytotoxicity was positively correlated with the expression level of PD-L1
(117)	2023	Thailand	Cell culture experimen	CCA	peptide vaccine	Peptide-pulsed DC-activated autologous HLA-A* 11:01-restricted T cells efficiently lysed KKU-213A (HLA-A*11:01) CCA cells compared to conventional tumor lysate-pulsed DC.
(121)	2019	China	clinical trial	CCA	allogenic γδ T cell immunotherapy	Allogeneic γδ T cell therapy positively modulated the peripheral blood immune function of the patients, reduced CCA tumor cell activity, and prolonged the life span of the patients.
(122)	2024	Thailand	Cell culture experimen	CCA	Vγ9Vδ2 T cells	Vγ9Vδ2 T cells can mediate cytotoxic effects on cholangiocarcinoma

## 7 Potential risks and side effects of immunotherapy for T-cell-associated cholangiocarcinoma

Potential risks and side effects are inherent in T-cell cholangiocarcinoma immunotherapy (129). These include Cytokine Release Syndrome (CRS), characterized by elevated levels of inflammatory cytokines, particularly interleukin (IL)6, due to immune activation (130). Symptoms range from high fever and flu-like symptoms to life-threatening complications such as organ failure (131). Neurotoxicity is another severe side effect, with patients potentially experiencing impaired consciousness, speech difficulties, balance loss, and in extreme cases, seizures, hallucinations, and coma (131–135).3. Tumor lysis syndrome (TLS) is a metabolic disorder caused by rapid tumor necrosis, resulting in conditions like hyperuricemia and hyperkalemia (136, 137). There’s also the risk of damage from attacks on normal tissues due to minimal expression of tumor-associated antigens (also known as off-target effect) (138). Therefore, enhancing the safety and efficacy of T-cell immunotherapy for cholangiocarcinoma is a significant challenge in cancer treatment.

## 8 Metabolic reprogramming of T cells in cholangiocarcinoma

A review of the literature reveals a limited number of studies focusing on the metabolic reprogramming of T cells in cholangiocarcinoma. However, research on other tumors has demonstrated that resting CD8^+^ T cells undergo dynamic shifts in metabolism, transitioning from oxidative metabolism to aerobic glycolysis upon activation. This transition is essential for supporting growth and differentiation into cytotoxic T cells, which can divide every 6–8 hours and produce inflammatory cytokines as well as cytolytic granules, including perforin and granzyme B (139). Tumor glucose consumption metabolically restricts T cells, leading to diminished mammalian target of rapamycin (mTOR) activity, reduced glycolytic capacity, and decreased IFN-γ production (140). For regulatory T (Treg) cells, the transcription factor Foxp3 reprograms T cell metabolism by suppressing Myc, a nuclear phosphoprotein involved in cell cycle progression, apoptosis, cellular transformation, and glycolysis. This reprogramming enhances mitochondrial oxidative phosphorylation (OXPHOS) and increases nicotinamide adenine dinucleotide oxidation (141). These adaptations confer a metabolic advantage to Tregs in low-glucose, lactate-rich environments. This metabolic phenotype may explain how Tregs promote peripheral immune tolerance during tissue injury and how cancer cells evade immune destruction in the tumor microenvironment. Thus, it is crucial to conduct studies targeting the metabolic reprogramming of T cells in cholangiocarcinoma.

## 9 Conclusion

From reading the literature published so far studying cholangiocarcinoma and T lymphocytes, we can assume that regardless of CCA subtype, CD8^+^ and CD4^+^ T cells are mainly located in the peri-tumor area, and Foxp3^+^ T cells mainly infiltrate in the tumor center, but for some The contrary reports may be related to different sample sizes and research methods, so more research is needed to confirm. We found that under the same conditions, PBC with chronic inflammation of the bile ducts with an autoimmune background are less susceptible to cholangiocarcinoma, which is related to their greater type 1 and type 2 T cell responses. The Fas/FasL signaling pathway and EGFR/PI3K/Akt signaling pathway related to T lymphocytes are involved in the progression of cholangiocarcinoma. I-FLICE, MUC1, MMP14, mIDH1, and SPP1 were found to be highly expressed in cholangiocarcinoma tissues, and they promoted cholangiocarcinoma progression by affecting T cell interactions or T cell immune infiltration. The more detailed mechanism remains to be elucidated, which has considerable potential for precise tumor treatment. Different immune cells and their subtypes have different prognostic effects on the long-term outcome of CCA. High levels of CD8^+^ T-cell infiltration in CCA are associated with a better prognosis, and high levels of Tex cells are associated with a poor prognosis. High density of CD4^+^ T cells at the tumor edge also seems to be associated with good DFS and OS. In contrast, a high number of Tregs is likely to be associated with worse OS. Future studies are definitely needed to elucidate the prognostic relevance of TILs in the long-term outcome of CCA. Currently, the main treatments for CCA include surgery and chemotherapy. Problems such as poor surgical results and chemotherapy resistance pose challenges to the treatment of cholangiocarcinoma. The availability of immunotherapies, including ICIs, cancer vaccines, and adoptive T-cell therapies, holds great potential to enable precision oncology treatment. But the side effects of immunotherapy also need to be taken seriously, and it is important to improve the safety of treatment for patients. Research on metabolic reprogramming of cholangiocarcinoma T cells also needs to be carried out.

## Glossary

CCA: Cholangiocarcinoma
eCCA: extrahepatic cholangiocarcinoma
iCCA: intrahepatic cholangiocarcinoma
DFS: disease-free survival
OS: overall survival
RFS: relapse-free survival
TTR: Time to recurrence
IT: intratumoral
PT: peritumoral
TM: tumor margin
IHC: Immunohistochemistry
mIHC: multiplexed immunohistochemistry
HE: hematoxylin-eosin staining
ELISA: Enzyme-Linked Immunosorbnent Assay
ScRNA: single-cell RNA sequencing
scTCR: single-cell T cell receptor
DC: dendritic cell
TILs: tumor-infiltrating lymphocytes
CAR T cells: chimeric antigen receptor‐T cells
CAR4 T cells: Fourth-generation chimeric antigen receptor (CAR4) T cells
KKU-213A: CCA cell lines
PD-1: programmed death ligand 1
TIM-3: T-cell Ig and mucin domain-3 protein
TIGIT: T-cell Ig and immunoreceptor tyrosine-based inhibitory motif domain
NKG2A: NK group 2 member A
CTLA-4: cytotoxic T lymphocyte antigen-4
CCR6: C-C chemokine receptor 6
CXCR3: C-X-C motif chemokine receptor 3
LAG-3: lymphocyte-activation gene 3
FOXP3: forkhead box P3
MMP: matrix metalloproteinases
FLICE: caspase 8
EGFR: Epidermal growth factor receptor
PI3K: Phosphatidylinositol-3 kinase
Akt: Protein kinase B
TET2: Tet Methylcytosine Dioxygenase 2
IFN-γ: interferon-γ
CXCL: chemokine [C-X-C motif] ligand 9
Tigit: T cell immunoglobulin and ITIM domain
Tim-3: T‐cell immunoglobulin and mucin domain‐3
TGFβ: Transforming Growth Factor Beta 1
scVF: single‐chain fragment variable
TP53: Tumor Protein P53
KRAS: KRAS Proto-Oncogene, GTPase
RNF43: Ring Finger Protein 43
HLA: Human leukocyte antigen.

## References

[1] Dadgar N Arunachalam AK Hong H Phoon YP Arpi-Palacios JE Uysal M. Advancing cholangiocarcinoma care: insights and innovations in T cell therapy. Cancers. (2024) 16(18):3232. doi: 10.3390/cancers16183232 39335203 PMC11429565

[2] Cadamuro M Stecca T Brivio S Mariotti V Fiorotto R Spirli C. The deleterious interplay between tumor epithelia and stroma in cholangiocarcinoma. Biochimica et biophysica acta Molecular basis of disease. Biochim Biophys Acta Mol Basis Dis. (2018) 1864(10):1435–43. doi: 10.1016/j.bbadis.2017.07.028 PMC6386155 28757170

[3] Razumilava N Gores GJ. Cholangiocarcinoma. Lancet (London England). (2014) 383:2168–79. doi: 10.1016/S0140-6736(13)61903-0 PMC4069226 24581682

[4] Goldaracena N Gorgen A Sapisochin G. Current status of liver transplantation for cholangiocarcinoma. Liver Transplantation: Off Publ Am Assoc Study Liver Dis Int Liver Transplant Society. (2018) 24:294–303. doi: 10.1002/lt.24955 29024405

[5] Lightner AL Dadgar N Vaidya A Simon R Fulmer C Siddiki H. Mesenchymal stem cells: A novel treatment option for primary sclerosing cholangitis. Cell Biol Int. (2023) 47:467–79. doi: 10.1002/cbin.11943 36321586

[6] Shin DW Moon SH Kim JH. Diagnosis of cholangiocarcinoma. Diagnostics (Basel Switzerland). (2023) 13(2):233. doi: 10.3390/diagnostics13020233 36673043 PMC9858255

[7] Al-Bahrani R Abuetabh Y Zeitouni N Sergi C. Cholangiocarcinoma: risk factors, environmental influences and oncogenesis. Ann Clin Lab Sci. (2013) 43(2):195–210. doi: 10.2141/2013.43.2.195 23694797

[8] Buettner S van Vugt JL Gani F Groot Koerkamp B Margonis GA Ethun CG. A comparison of prognostic schemes for perihilar cholangiocarcinoma. J Gastrointestinal Surgery: Off J Soc Surg Alimentary Tract. (2016) 20:1716–24. doi: 10.1007/s11605-016-3203-2 PMC5450027 27412318

[9] Cao H Huang T Dai M Kong X Liu H Zheng Z. Tumor microenvironment and its implications for antitumor immunity in cholangiocarcinoma: future perspectives for novel therapies. Int J Biol Sci. (2022) 18:5369–90. doi: 10.7150/ijbs.73949 PMC9461676 36147461

[10] Kelley RK Ueno M Yoo C Finn RS Furuse J Ren Z. Pembrolizumab in combination with gemcitabine and cisplatin compared with gemcitabine and cisplatin alone for patients with advanced biliary tract cancer (KEYNOTE-966): a randomised, double-blind, placebo-controlled, phase 3 trial. Lancet (London England). (2023) 401:1853–65. doi: 10.1016/S0140-6736(23)00727-4 37075781

[11] Rimassa L Personeni N Aghemo A Lleo A. The immune milieu of cholangiocarcinoma: From molecular pathogenesis to precision medicine. J Autoimmun. (2019) 100:17–26. doi: 10.1016/j.jaut.2019.03.007 30862450

[12] Liu D Heij LR Czigany Z Dahl E Lang SA Ulmer TF. The role of tumor-infiltrating lymphocytes in cholangiocarcinoma. J Exp Clin Cancer Res: CR. (2022) 41:127. doi: 10.1186/s13046-022-02340-2 35392957 PMC8988317

[13] Kumar BV Connors TJ Farber DL. Human T cell development, localization, and function throughout life. Immunity. (2018) 48:202–13. doi: 10.1016/j.immuni.2018.01.007 PMC5826622 29466753

[14] Ruterbusch M Pruner KB Shehata L Pepper M. *In vivo* CD4(+) T cell differentiation and function: revisiting the th1/th2 paradigm. Annu Rev Immunol. (2020) 38:705–25. doi: 10.1146/annurev-immunol-103019-085803 32340571

[15] Geginat J Paroni M Maglie S Alfen JS Kastirr I Gruarin P. Plasticity of human CD4 T cell subsets. Front Immunol. (2014) 5:630. doi: 10.3389/fimmu.2014.00630 25566245 PMC4267263

[16] Ascierto ML Kmieciak M Idowu MO Manjili R Zhao Y Grimes M. A signature of immune function genes associated with recurrence-free survival in breast cancer patients. Breast Cancer Res Treat. (2012) 131:871–80. doi: 10.1007/s10549-011-1470-x PMC3431022 21479927

[17] Tosolini M Kirilovsky A Mlecnik B Fredriksen T Mauger S Bindea G. Clinical impact of different classes of infiltrating T cytotoxic and helper cells (Th1, th2, treg, th17) in patients with colorectal cancer. Cancer Res. (2011) 71:1263–71. doi: 10.1158/0008-5472.CAN-10-2907 21303976

[18] Mann GJ Pupo GM Campain AE Carter CD Schramm SJ Pianova S. BRAF mutation, NRAS mutation, and the absence of an immune-related expressed gene profile predict poor outcome in patients with stage III melanoma. J Invest Dermatol. (2013) 133:509–17. doi: 10.1038/jid.2012.283 22931913

[19] Curtis C Shah SP Chin SF Turashvili G Rueda OM Dunning MJ. The genomic and transcriptomic architecture of 2,000 breast tumours reveals novel subgroups. Nature. (2012) 486:346–52. doi: 10.1038/nature10983 PMC3440846 22522925

[20] Leffers N Fehrmann RS Gooden MJ Schulze UR Ten Hoor KA Hollema H. Identification of genes and pathways associated with cytotoxic T lymphocyte infiltration of serous ovarian cancer. Br J Cancer. (2010) 103:685–92. doi: 10.1038/sj.bjc.6605820 PMC2938262 20664601

[21] Laheurte C Dosset M Vernerey D Boullerot L Gaugler B Gravelin E. Distinct prognostic value of circulating anti-telomerase CD4(+) Th1 immunity and exhausted PD-1(+)/TIM-3(+) T cells in lung cancer. Br J Cancer. (2019) 121:405–16. doi: 10.1038/s41416-019-0531-5 PMC6738094 31358938

[22] Xu X Wang R Su Q Huang H Zhou P Luan J. Expression of Th1- Th2- and Th17-associated cytokines in laryngeal carcinoma. Oncol Lett. (2016) 12:1941–8. doi: 10.3892/ol.2016.4854 PMC4998098 27588143

[23] Dengel LT Norrod AG Gregory BL Clancy-Thompson E Burdick MD Strieter RM. Interferons induce CXCR3-cognate chemokine production by human metastatic melanoma. J Immunother (Hagerstown Md: 1997). (2010) 33:965–74. doi: 10.1097/CJI.0b013e3181fb045d PMC3110268 20948440

[24] House IG Savas P Lai J Chen AXY Oliver AJ Teo ZL. Macrophage-derived CXCL9 and CXCL10 are required for antitumor immune responses following immune checkpoint blockade. Clin Cancer Res: An Off J Am Assoc Cancer Res. (2020) 26:487–504. doi: 10.1158/1078-0432.CCR-19-1868 31636098

[25] Bos R Sherman LA. CD4+ T-cell help in the tumor milieu is required for recruitment and cytolytic function of CD8+ T lymphocytes. Cancer Res. (2010) 70:8368–77. doi: 10.1158/0008-5472.CAN-10-1322 PMC2970736 20940398

[26] Shankaran V Ikeda H Bruce AT White JM Swanson PE Old LJ. IFNgamma and lymphocytes prevent primary tumour development and shape tumour immunogenicity. Nature. (2001) 410:1107–11. doi: 10.1038/35074122 11323675

[27] Tepper RI Coffman RL Leder P. An eosinophil-dependent mechanism for the antitumor effect of interleukin-4. Sci (New York NY). (1992) 257:548–51. doi: 10.1126/science.1636093 1636093

[28] Lorvik KB Hammarström C Fauskanger M Haabeth OA Zangani M Haraldsen G. Adoptive transfer of tumor-specific th2 cells eradicates tumors by triggering an *in situ* inflammatory immune response. Cancer Res. (2016) 76:6864–76. doi: 10.1158/0008-5472.CAN-16-1219 27634753

[29] Rodriguez-Tirado C Entenberg D Li J Qian BZ Condeelis JS Pollard JW. Interleukin 4 controls the pro-tumoral role of macrophages in mammary cancer pulmonary metastasis in mice. Cancers. (2022) 14(17):4336. doi: 10.3390/cancers14174336 36077870 PMC9454655

[30] Lazarski CA Ford J Katzman SD Rosenberg AF Fowell DJ. IL-4 attenuates Th1-associated chemokine expression and Th1 trafficking to inflamed tissues and limits pathogen clearance. PloS One. (2013) 8:e71949. doi: 10.1371/journal.pone.0071949 23991011 PMC3753298

[31] Boieri M Malishkevich A Guennoun R Marchese E Kroon S Trerice KE. CD4+ T helper 2 cells suppress breast cancer by inducing terminal differentiation. J Exp Med. (2022) 219:e20201963. doi: 10.1084/jem.20201963 35657353 PMC9170526

[32] Johansson M Denardo DG Coussens LM. Polarized immune responses differentially regulate cancer development. Immunol Rev. (2008) 222:145–54. doi: 10.1111/j.1600-065X.2008.00600.x PMC2494984 18363999

[33] Plitas G Rudensky AY. Regulatory T cells: differentiation and function. Cancer Immunol Res. (2016) 4:721–5. doi: 10.1158/2326-6066.CIR-16-0193 PMC5026325 27590281

[34] Kanamori M Nakatsukasa H Okada M Lu Q Yoshimura A. Induced regulatory T cells: their development, stability, and applications. Trends Immunol. (2016) 37:803–11. doi: 10.1016/j.it.2016.08.012 27623114

[35] Raffin C Vo LT Bluestone JA. T(reg) cell-based therapies: challenges and perspectives. Nat Rev Immunol. (2020) 20:158–72. doi: 10.1038/s41577-019-0232-6 PMC7814338 31811270

[36] McRitchie BR Akkaya B. Exhaust the exhausters: Targeting regulatory T cells in the tumor microenvironment. Front Immunol. (2022) 13:940052. doi: 10.3389/fimmu.2022.940052 36248808 PMC9562032

[37] Kaech SM Wherry EJ. Heterogeneity and cell-fate decisions in effector and memory CD8+ T cell differentiation during viral infection. Immunity. (2007) 27:393–405. doi: 10.1016/j.immuni.2007.08.007 17892848 PMC3431921

[38] Joshi NS Cui W Chandele A Lee HK Urso DR Hagman J. Inflammation directs memory precursor and short-lived effector CD8(+) T cell fates via the graded expression of T-bet transcription factor. Immunity. (2007) 27:281–95. doi: 10.1016/j.immuni.2007.07.010 PMC2034442 17723218

[39] Kaech SM Tan JT Wherry EJ Konieczny BT Surh CD Ahmed R. Selective expression of the interleukin 7 receptor identifies effector CD8 T cells that give rise to long-lived memory cells. Nat Immunol. (2003) 4:1191–8. doi: 10.1038/ni1009 14625547

[40] Wherry EJ Blattman JN Murali-Krishna K van der Most R Ahmed R. Viral persistence alters CD8 T-cell immunodominance and tissue distribution and results in distinct stages of functional impairment. J Virol. (2003) 77:4911–27. doi: 10.1128/JVI.77.8.4911-4927.2003 PMC152117 12663797

[41] Dolina JS Van-Braeckel-Budimir N Thomas GD Salek-Ardakani S. CD8(+) T cell exhaustion in cancer. Front Immunol. (2021) 12:715234. doi: 10.3389/fimmu.2021.715234 34354714 PMC8330547

[42] Kurachi M. CD8(+) T cell exhaustion. Semin Immunopathol. (2019) 41:327–37. doi: 10.1007/s00281-019-00744-5 30989321

[43] Oliveira G Wu CJ. Dynamics and specificities of T cells in cancer immunotherapy. Nat Rev Cancer. (2023) 23:295–316. doi: 10.1038/s41568-023-00560-y 37046001 PMC10773171

[44] Heij L Bednarsch J Tan X Rosin M Appinger S Reichel K. Expression of checkpoint molecules in the tumor microenvironment of intrahepatic cholangiocarcinoma: implications for immune checkpoint blockade therapy. Cells. (2023) 12(6):851. doi: 10.3390/cells12060851 36980192 PMC10047585

[45] Rizzo A Ricci AD Brandi G. PD-L1, TMB, MSI, and other predictors of response to immune checkpoint inhibitors in biliary tract cancer. Cancers. (2021) 13(3):558. doi: 10.3390/cancers13030558 33535621 PMC7867133

[46] Thommen DS Schreiner J Müller P Herzig P Roller A Belousov A. Progression of lung cancer is associated with increased dysfunction of T cells defined by coexpression of multiple inhibitory receptors. Cancer Immunol Res. (2015) 3:1344–55. doi: 10.1158/2326-6066.CIR-15-0097 26253731

[47] Penaloza-MacMaster P Ur Rasheed A Iyer SS Yagita H Blazar BR Ahmed R. Opposing effects of CD70 costimulation during acute and chronic lymphocytic choriomeningitis virus infection of mice. J Virol. (2011) 85:6168–74. doi: 10.1128/JVI.02205-10 PMC3126534 21507976

[48] Esensten JH Helou YA Chopra G Weiss A Bluestone JA. CD28 costimulation: from mechanism to therapy. Immunity. (2016) 44:973–88. doi: 10.1016/j.immuni.2016.04.020 PMC4932896 27192564

[49] Hui E Cheung J Zhu J Su X Taylor MJ Wallweber HA. T cell costimulatory receptor CD28 is a primary target for PD-1-mediated inhibition. Sci (New York NY). (2017) 355:1428–33. doi: 10.1126/science.aaf1292 PMC6286077 28280247

[50] Chen L Sun R Xu J Zhai W Zhang D Yang M. Tumor-derived IL33 promotes tissue-resident CD8(+) T cells and is required for checkpoint blockade tumor immunotherapy. Cancer Immunol Res. (2020) 8:1381–92. doi: 10.1158/2326-6066.CIR-19-1024 PMC7642190 32917659

[51] Hoffmann JC Schön MP. Integrin α(E)(CD103)β(7) in epithelial cancer. Cancers. (2021) 13(24):6211. doi: 10.3390/cancers13246211 34944831 PMC8699740

[52] Mueller SN Mackay LK. Tissue-resident memory T cells: local specialists in immune defence. Nat Rev Immunol. (2016) 16:79–89. doi: 10.1038/nri.2015.3 26688350

[53] Chen L Huang H Huang Z Chen J Liu Y Wu Y. Prognostic values of tissue-resident CD8(+)T cells in human hepatocellular carcinoma and intrahepatic cholangiocarcinoma. World J Surg Oncol. (2023) 21:124. doi: 10.1186/s12957-023-03009-6 37024870 PMC10077621

[54] Zhou ZQ Zhang Y Xu ZY Tang XL Chen XH Guan J. Dissecting cellular heterogeneity and intercellular communication in cholangiocarcinoma: implications for individualized therapeutic strategies. Front Genet. (2023) 14:1241834. doi: 10.3389/fgene.2023.1241834 38239853 PMC10794609

[55] Zhang QW Zhu MX Liu WF Rui WW Chen Y Ding XY. Identification of clinically relevant subsets CD39(+)PD-1(+)CD8(+) T cells and CD39(+) regulatory T cells in intrahepatic cholangiocarcinoma using single-cell CyTOF. Trans Oncol. (2024) 44:101954. doi: 10.1016/j.tranon.2024.101954 PMC11024660 38608405

[56] Shi H Li Z Zhu M. Circulating immune cells predict prognosis and clinical response to chemotherapy in cholangiocarcinoma. Curr Medicinal Chem. (2024) 73:6618–24. doi: 10.2174/0109298673296618240424095548 38698750

[57] Ji GW Xu Q Jiao CY Lu M Xu ZG Zhang B. Translating imaging traits of mass-forming intrahepatic cholangiocarcinoma into the clinic: From prognostic to therapeutic insights. JHEP Reports: Innovation Hepatol. (2023) 5:100839. doi: 10.1016/j.jhepr.2023.100839 PMC10468367 37663120

[58] Alvisi G Termanini A Soldani C Portale F Carriero R Pilipow K. Multimodal single-cell profiling of intrahepatic cholangiocarcinoma defines hyperactivated Tregs as a potential therapeutic target. J Hepatol. (2022) 77:1359–72. doi: 10.1016/j.jhep.2022.05.043 35738508

[59] Kim HD Kim JH Ryu YM Kim D Lee S Shin J. Spatial distribution and prognostic implications of tumor-infiltrating foxP3- CD4+ T cells in biliary tract cancer. Cancer Res Treat. (2021) 53:162–71. doi: 10.4143/crt.2020.704 PMC7812013 32878426

[60] Kim HD Jeong S Park S Lee YJ Ju YS Kim D. Implication of CD69(+) CD103(+) tissue-resident-like CD8(+) T cells as a potential immunotherapeutic target for cholangiocarcinoma. Liver International: Off J Int Assoc Study Liver. (2021) 41:764–76. doi: 10.1111/liv.14814 33548061

[61] Yuan Y. Spatial heterogeneity in the tumor microenvironment. Cold Spring Harbor Perspect Med. (2016) 6(8):a026583. doi: 10.1101/cshperspect.a026583 PMC4968167 27481837

[62] Heindl A Nawaz S Yuan Y. Mapping spatial heterogeneity in the tumor microenvironment: a new era for digital pathology. Lab Investigation J Tech Methods Pathol. (2015) 95:377–84. doi: 10.1038/labinvest.2014.155 25599534

[63] Kather JN Suarez-Carmona M Charoentong P Weis CA Hirsch D Bankhead P. Topography of cancer-associated immune cells in human solid tumors. eLife. (2018) 7:e36967. doi: 10.7554/eLife.36967 30179157 PMC6133554

[64] Chen DS Mellman I. Elements of cancer immunity and the cancer-immune set point. Nature. (2017) 541:321–30. doi: 10.1038/nature21349 28102259

[65] Deng Q Luo Y Chang C Wu H Ding Y Xiao R. The emerging epigenetic role of CD8+T cells in autoimmune diseases: A systematic review. Front Immunol. (2019) 10:856. doi: 10.3389/fimmu.2019.00856 31057561 PMC6482221

[66] Paillet J Plantureux C Lévesque S Le Naour J Stoll G Sauvat A. Autoimmunity affecting the biliary tract fuels the immunosurveillance of cholangiocarcinoma. J Exp Med. (2021) 218(10):e20200853. doi: 10.1084/jem.20200853 34495298 PMC8429038

[67] Carnevale G Carpino G Cardinale V Pisciotta A Riccio M Bertoni L. Activation of Fas/FasL pathway and the role of c-FLIP in primary culture of human cholangiocarcinoma cells. Sci Rep. (2017) 7:14419. doi: 10.1038/s41598-017-14838-3 29089545 PMC5663931

[68] Li Z Zhang L Zou S. The “Fas counterattack”: a mechanism for immune evasion in human hilar cholangiocarcinomas. Zhonghua Yi Xue Za Zhi. (2002) 82(5):606–9. doi: 10.3761/j.issn.0376-2491.2002.05.028 12133481

[69] Que FG Phan VA Phan VH Celli A Batts K LaRusso NF. Cholangiocarcinomas express Fas ligand and disable the Fas receptor. Hepatol (Baltimore Md). (1999) 30:1398–404. doi: 10.1002/hep.510300618 10573518

[70] Zhang G Zheng G Zhang H Qiu L. MUC1 induces the accumulation of Foxp3(+) Treg cells in the tumor microenvironment to promote the growth and metastasis of cholangiocarcinoma through the EGFR/PI3K/Akt signaling pathway. Int Immunopharmacol. (2023) 118:110091. doi: 10.1016/j.intimp.2023.110091 37018979

[71] Claesson-Welsh L. How the matrix metalloproteinase MMP14 contributes to the progression of colorectal cancer. J Clin Invest. (2020) 130:1093–5. doi: 10.1172/JCI135239 PMC7269590 32015228

[72] Wu J Guo Y Zuo ZF Zhu ZW Han L. MMP14 is a diagnostic gene of intrahepatic cholangiocarcinoma associated with immune cell infiltration. World J Gastroenterol. (2023) 29:2961–78. doi: 10.3748/wjg.v29.i19.2961 PMC10237093 37274806

[73] Borger DR Tanabe KK Fan KC Lopez HU Fantin VR Straley KS. Frequent mutation of isocitrate dehydrogenase (IDH)1 and IDH2 in cholangiocarcinoma identified through broad-based tumor genotyping. Oncol. (2012) 17:72–9. doi: 10.1634/theoncologist.2011-0386 PMC3267826 22180306

[74] Lowery MA Ptashkin R Jordan E Berger MF Zehir A Capanu M. Comprehensive molecular profiling of intrahepatic and extrahepatic cholangiocarcinomas: potential targets for intervention. Clin Cancer Res: An Off J Am Assoc Cancer Res. (2018) 24:4154–61. doi: 10.1158/1078-0432.CCR-18-0078 PMC6642361 29848569

[75] Waitkus MS Diplas BH Yan H. Biological role and therapeutic potential of IDH mutations in cancer. Cancer Cell. (2018) 34:186–95. doi: 10.1016/j.ccell.2018.04.011 PMC6092238 29805076

[76] Wu MJ Shi L Dubrot J Merritt J Vijay V Wei TY. Mutant IDH inhibits IFNγ-TET2 signaling to promote immunoevasion and tumor maintenance in cholangiocarcinoma. Cancer Discov. (2022) 12:812–35. doi: 10.1158/2159-8290.CD-21-1077 PMC8904298 34848557

[77] Zhu Y Kwong LN. IDH1 inhibition reawakens the immune response against cholangiocarcinoma. Cancer Discov. (2022) 12:604–5. doi: 10.1158/2159-8290.CD-21-1643 35257150

[78] Klement JD Paschall AV Redd PS Ibrahim ML Lu C Yang D. An osteopontin/CD44 immune checkpoint controls CD8+ T cell activation and tumor immune evasion. J Clin Invest. (2018) 128:5549–60. doi: 10.1172/JCI123360 PMC6264631 30395540

[79] Cheng M Liang G Yin Z Lin X Sun Q Liu Y. Immunosuppressive role of SPP1-CD44 in the tumor microenvironment of intrahepatic cholangiocarcinoma assessed by single-cell RNA sequencing. J Cancer Res Clin Oncol. (2023) 149:5497–512. doi: 10.1007/s00432-022-04498-w PMC11797745 36469154

[80] Xu YP Zhou YQ Zhao YJ Zhao Y Wang F Huang XY. High level of CD73 predicts poor prognosis of intrahepatic cholangiocarcinoma. J Cancer. (2021) 12:4655–60. doi: 10.7150/jca.51038 PMC8210563 34149929

[81] Kitano Y Okabe H Yamashita YI Nakagawa S Saito Y Umezaki N. Tumour-infiltrating inflammatory and immune cells in patients with extrahepatic cholangiocarcinoma. Br J Cancer. (2018) 118:171–80. doi: 10.1038/bjc.2017.401 PMC5785749 29123259

[82] Xia T Li K Niu N Shao Y Ding D Thomas DL. Immune cell atlas of cholangiocarcinomas reveals distinct tumor microenvironments and associated prognoses. J Hematol Oncol. (2022) 15:37. doi: 10.1186/s13045-022-01253-z 35346322 PMC8962046

[83] Wirta EV Szeto S Koppatz H Nordin A Mäkisalo H Arola J. High immune cell infiltration predicts improved survival in cholangiocarcinoma. Front Oncol. (2024) 14:1333926. doi: 10.3389/fonc.2024.1333926 38751812 PMC11094285

[84] Tian L Ma J Ma L Zheng B Liu L Song D. PD-1/PD-L1 expression profiles within intrahepatic cholangiocarcinoma predict clinical outcome. World J Surg Oncol. (2020) 18:303. doi: 10.1186/s12957-020-02082-5 33228682 PMC7686719

[85] Kansy BA Concha-Benavente F Srivastava RM Jie HB Shayan G Lei Y. PD-1 status in CD8(+) T cells associates with survival and anti-PD-1 therapeutic outcomes in head and neck cancer. Cancer Res. (2017) 77:6353–64. doi: 10.1158/0008-5472.CAN-16-3167 PMC5690836 28904066

[86] Zheng Y Huang N Kuang S Zhang J Zhao H Wu J. The clinicopathological significance and relapse predictive role of tumor microenvironment of intrahepatic cholangiocarcinoma after radical surgery. Cancer. (2023) 129:393–404. doi: 10.1002/cncr.v129.3 36433731 PMC10099237

[87] Konishi D Umeda Y Yoshida K Shigeyasu K Yano S Toji T. Regulatory T cells induce a suppressive immune milieu and promote lymph node metastasis in intrahepatic cholangiocarcinoma. Br J Cancer. (2022) 127:757–65. doi: 10.1038/s41416-022-01838-y PMC9381563 35597869

[88] Miyara M Yoshioka Y Kitoh A Shima T Wing K Niwa A. Functional delineation and differentiation dynamics of human CD4+ T cells expressing the FoxP3 transcription factor. Immunity. (2009) 30:899–911. doi: 10.1016/j.immuni.2009.03.019 19464196

[89] Wang L Simons DL Lu X Tu TY Solomon S Wang R. Connecting blood and intratumoral T(reg) cell activity in predicting future relapse in breast cancer. Nat Immunol. (2019) 20:1220–30. doi: 10.1038/s41590-019-0429-7 PMC8802768 31285626

[90] Shi JY Ma LJ Zhang JW Duan M Ding ZB Yang LX. FOXP3 Is a HCC suppressor gene and Acts through regulating the TGF-β/Smad2/3 signaling pathway. BMC Cancer. (2017) 17:648. doi: 10.1186/s12885-017-3633-6 28903735 PMC5598072

[91] Chłopik A Selim MA Peng Y Wu CL Tell-Marti G Paral KM. Prognostic role of tumoral PDL1 expression and peritumoral FoxP3+ lymphocytes in vulvar melanomas. Hum Pathol. (2018) 73:176–83. doi: 10.1016/j.humpath.2017.12.022 29307625

[92] Kida A Mizukoshi E Kido H Toyama T Terashima T Arai K. The characteristics of the immune cell profiles in peripheral blood in cholangiocarcinoma patients. Hepatol Int. (2021) 15:695–706. doi: 10.1007/s12072-021-10177-8 33754279

[93] Zimmer CL Filipovic I Cornillet M O’Rourke CJ Berglin L Jansson H. Mucosal-associated invariant T-cell tumor infiltration predicts long-term survival in cholangiocarcinoma. Hepatol (Baltimore Md). (2022) 75:1154–68. doi: 10.1002/hep.32222 34719787

[94] Perkhofer L Beutel AK Ettrich TJ. Immunotherapy: pancreatic cancer and extrahepatic biliary tract cancer. Visceral Med. (2019) 35:28–37. doi: 10.1159/000497291 PMC6597904 31312647

[95] Sanmamed MF Chen L. Inducible expression of B7-H1 (PD-L1) and its selective role in tumor site immune modulation. Cancer J (Sudbury Mass). (2014) 20:256–61. doi: 10.1097/PPO.0000000000000061 PMC4455021 25098285

[96] Zou W Chen L. Inhibitory B7-family molecules in the tumour microenvironment. Nat Rev Immunol. (2008) 8:467–77. doi: 10.1038/nri2326 18500231

[97] Wathikthinnakon M Luangwattananun P Sawasdee N Chiawpanit C Lee VS Nimmanpipug P. Combination gemcitabine and PD-L1xCD3 bispecific T cell engager (BiTE) enhances T lymphocyte cytotoxicity against cholangiocarcinoma cells. Sci Rep. (2022) 12:6154. doi: 10.1038/s41598-022-09964-6 35418130 PMC9007942

[98] Stevanović S Pasetto A Helman SR Gartner JJ Prickett TD Howie B. Landscape of immunogenic tumor antigens in successful immunotherapy of virally induced epithelial cancer. Science. (2017) 356(6334):200–5. doi: 10.1126/science.aak9510 PMC6295311 28408606

[99] Tran E Robbins PF Lu Y-C Prickett TD Gartner JJ Jia L. T-cell transfer therapy targeting mutant KRAS in cancer. N Engl J Med. (2016) 375(23):2255–62. doi: 10.1056/NEJMoa1609279 PMC5178827 27959684

[100] Martin SD Wick DA Nielsen JS Little N Holt RA Nelson BHJO. A library-based screening method identifies neoantigen-reactive T cells in peripheral blood prior to relapse of ovarian cancer. Oncoimmunology. (2018) 7(1):e1371895. doi: 10.1080/2162402X.2017.1371895 PMC5739566 29296522

[101] Pang Y Hou X Yang C Liu Y Jiang GJM. Advances on chimeric antigen receptor-modified T-cell therapy for oncotherapy. Mol Cancer. (2018) 17(1):1–10. doi: 10.1186/s12943-018-0840-y 29769134 PMC5956614

[102] Feng KC Guo YL Liu Y Dai HR Wang Y Lv HY. Cocktail treatment with EGFR-specific and CD133-specific chimeric antigen receptor-modified T cells in a patient with advanced cholangiocarcinoma. J Hematol Oncol. (2017) 10:4. doi: 10.1186/s13045-016-0378-7 28057014 PMC5217546

[103] Sangsuwannukul T Supimon K Sujjitjoon J Phanthaphol N Chieochansin T Poungvarin N. Anti-tumour effect of the fourth-generation chimeric antigen receptor T cells targeting CD133 against cholangiocarcinoma cells. Int Immunopharmacol. (2020) 89:107069. doi: 10.1016/j.intimp.2020.107069 33242709

[104] Soejima Y Takeuchi M Akashi T Sawabe M Fukusato T. β4 and β6 integrin expression is associated with the subclassification and clinicopathological features of intrahepatic cholangiocarcinoma. Int J Mol Sci. (2018) 19(4):1004. doi: 10.3390/ijms19041004 29584696 PMC5979350

[105] Sun Q Dong X Shang Y Sun F Niu J Li F. Integrin αvβ6 predicts poor prognosis and promotes resistance to cisplatin in hilar cholangiocarcinoma. Pathol Res Practice. (2020) 216:153022. doi: 10.1016/j.prp.2020.153022 32534716

[106] Supimon K Sangsuwannukul T Sujjitjoon J Phanthaphol N Chieochansin T Poungvarin N. Anti-mucin 1 chimeric antigen receptor T cells for adoptive T cell therapy of cholangiocarcinoma. Sci Rep. (2021) 11:6276. doi: 10.1038/s41598-021-85747-9 33737613 PMC7973425

[107] Phanthaphol N Somboonpatarakun C Suwanchiwasiri K Chieochansin T Sujjitjoon J Wongkham S. Chimeric antigen receptor T cells targeting integrin αvβ6 expressed on cholangiocarcinoma cells. Front Oncol. (2021) 11:657868. doi: 10.3389/fonc.2021.657868 33763382 PMC7982884

[108] Mao L Su S Li J Yu S Gong Y Chen C. Development of engineered CAR T cells targeting tumor-associated glycoforms of MUC1 for the treatment of intrahepatic cholangiocarcinoma. J Immunother (Hagerstown Md: 1997). (2023) 46:89–95. doi: 10.1097/CJI.0000000000000460 PMC9988215 36883998

[109] Suwanchiwasiri K Phanthaphol N Somboonpatarakun C Yuti P Sujjitjoon J Luangwattananun P. Bispecific T cell engager-armed T cells targeting integrin ανβ6 exhibit enhanced T cell redirection and antitumor activity in cholangiocarcinoma. Biomed Pharmacother. (2024) 175:116718. doi: 10.1016/j.biopha.2024.116718 38744221

[110] Supimon K Sangsuwannukul T Sujjitjoon J Chieochansin T Junking M Yenchitsomanus PT. Cytotoxic activity of anti-mucin 1 chimeric antigen receptor T cells expressing PD-1-CD28 switch receptor against cholangiocarcinoma cells. Cytotherapy. (2023) 25:148–61. doi: 10.1016/j.jcyt.2022.10.006 36396553

[111] Qiao Y Chen J Wang X Yan S Tan J Xia B. Enhancement of CAR-T cell activity against cholangiocarcinoma by simultaneous knockdown of six inhibitory membrane proteins. Cancer Commun (London England). (2023) 43:788–807. doi: 10.1002/cac2.12452 PMC10354409 37282786

[112] Zhang Y Chen S Li J Dai W Qian Y. Immune infiltrating cells in cholangiocarcinoma may become clinical diagnostic markers: based on bioinformatics analysis. World J Surg Oncol. (2021) 19:59. doi: 10.1186/s12957-021-02168-8 33618734 PMC7901112

[113] Wang J Loeuillard E Gores GJ Ilyas SI. Cholangiocarcinoma: what are the most valuable therapeutic targets - cancer-associated fibroblasts, immune cells, or beyond T cells? Expert Opin Ther Targets. (2021) 25(10):835–45. doi: 10.1080/14728222.2021.2010046 PMC9239407 34806500

[114] Jiraviriyakul A Songjang W Kaewthet P Tanawatkitichai P Bayan P Pongcharoen S. Honokiol-enhanced cytotoxic T lymphocyte activity against cholangiocarcinoma cells mediated by dendritic cells pulsed with damage-associated molecular patterns. World J Gastroenterol. (2019) 25(29):3941. doi: 10.3748/wjg.v25.i29.3941 31413529 PMC6689815

[115] Panya A Thepmalee C Sawasdee N Sujjitjoon J Phanthaphol N Junking M. Cytotoxic activity of effector T cells against cholangiocarcinoma is enhanced by self-differentiated monocyte-derived dendritic cells. Cancer Immunol Immunother. (2018) 67(10):1579–88. doi: 10.1007/s00262-018-2212-2 PMC11028072 30056600

[116] Junking M Grainok J Thepmalee C Wongkham S Yenchitsomanus P. Enhanced cytotoxic activity of effector T-cells against cholangiocarcinoma by dendritic cells pulsed with pooled mRNA. Tumour Biol. (2017) 39(10):1010428317733367. doi: 10.1177/1010428317733367 29034817

[117] Panya A Thepmalee C Sawasdee N Saengmuang S Luangwattananun P Yenchitsomanus PT. Enhancing cholangiocarcinoma immunotherapy with adoptive T cells targeting HLA-restricted neoantigen peptides derived from driver gene mutations. Biomed Pharmacother. (2023) 168:115827. doi: 10.1016/j.biopha.2023.115827 37939617

[118] Mittal D Gubin MM Schreiber RD Smyth MJ. New insights into cancer immunoediting and its three component phases—elimination, equilibrium and escape. Curr Opin Immunol. (2014) 27:16–25. doi: 10.1016/j.coi.2014.01.004 24531241 PMC4388310

[119] Silva-Santos B Mensurado S Coffelt SB. [amp]]gamma;δ T cells: pleiotropic immune effectors with therapeutic potential in cancer. Nat Rev Cancer. (2019) 19(7):392–404. doi: 10.1038/s41568-019-0153-5 31209264 PMC7614706

[120] Saura-Esteller J De Jong M King LA Ensing E Winograd B De Gruijl TD. Gamma delta T-cell based cancer immunotherapy: past-present-future. Front Immunol. (2022) 13:915837. doi: 10.3389/fimmu.2022.915837 35784326 PMC9245381

[121] Alnaggar M Xu Y Li J He J Chen J Li M. Allogenic Vγ9Vδ2 T cell as new potential immunotherapy drug for solid tumor: a case study for cholangiocarcinoma. J Immunother Cancer. (2019) 7:36. doi: 10.1186/s40425-019-0501-8 30736852 PMC6368763

[122] Sawaisorn P Gaballa A Saimuang K Leepiyasakulchai C Lertjuthaporn S Hongeng S. Human Vγ9Vδ2 T cell expansion and their cytotoxic responses against cholangiocarcinoma. Sci Rep. (2024) 14:1291. doi: 10.1038/s41598-024-51794-1 38221530 PMC10788337

[123] Gao Y Yang W Pan M Scully E Girardi M Augenlicht LH. Gamma delta T cells provide an early source of interferon gamma in tumor immunity. J Exp Med. (2003) 198:433–42. doi: 10.1084/jem.20030584 PMC2194096 12900519

[124] Xiang Z Tu W. Dual face of Vγ9Vδ2-T cells in tumor immunology: anti- versus pro-tumoral activities. Front Immunol. (2017) 8:1041. doi: 10.3389/fimmu.2017.01041 28894450 PMC5581348

[125] Chitadze G Oberg HH Wesch D Kabelitz D. The ambiguous role of γδ T lymphocytes in antitumor immunity. Trends Immunol. (2017) 38:668–78. doi: 10.1016/j.it.2017.06.004 28709825

[126] Hanahan D Weinberg RA. Hallmarks of cancer: the next generation. Cell. (2011) 144(5):646–74. doi: 10.1016/j.cell.2011.02.013 21376230

[127] Girardi M Oppenheim DE Steele CR Lewis JM Glusac E Filler R. Regulation of cutaneous Malignancy by γδ T cells. Science. (2001) 294(5542):605–9. doi: 10.1126/science.1063916 11567106

[128] Almeida AR Correia DV Fernandes-Platzgummer A da Silva CL da Silva MG Anjos DR. Delta one T cells for immunotherapy of chronic lymphocytic leukemia: clinical-grade expansion/differentiation and preclinical proof of concept. Clin Cancer Res. (2016) 22(23):5795–804. doi: 10.1158/1078-0432.CCR-16-0597 27307596

[129] The Lancet O. Immunotherapy: balancing the risks and benefits. Lancet Oncol. (2024) 25:147. doi: 10.1016/S1470-2045(24)00028-7 38301683

[130] Lee DW Gardner R Porter DL Louis CU Ahmed N Jensen M. Current concepts in the diagnosis and management of cytokine release syndrome. Blood. (2014) 124(2):188–95. doi: 10.1182/blood-2014-05-552729 PMC4093680 24876563

[131] Ramos CA Savoldo B Torrano V Ballard B Zhang H Dakhova O. Clinical responses with T lymphocytes targeting Malignancy-associated κ light chains. J Clin Invest. (2016) 126:2588–96. doi: 10.1172/JCI86000 PMC4922690 27270177

[132] Lee DW Kochenderfer JN Stetler-Stevenson M Cui YK Delbrook C Feldman SA. T cells expressing CD19 chimeric antigen receptors for acute lymphoblastic leukaemia in children and young adults: a phase 1 dose-escalation trial. Lancet (London England). (2015) 385:517–28. doi: 10.1016/S0140-6736(14)61403-3 PMC7065359 25319501

[133] Maude SL Frey N Shaw PA Aplenc R Barrett DM Bunin NJ. Chimeric antigen receptor T cells for sustained remissions in leukemia. New Engl J Med. (2014) 371:1507–17. doi: 10.1056/NEJMoa1407222 PMC4267531 25317870

[134] Kochenderfer JN Dudley ME Kassim SH Somerville RP Carpenter RO Stetler-Stevenson M. Chemotherapy-refractory diffuse large B-cell lymphoma and indolent B-cell Malignancies can be effectively treated with autologous T cells expressing an anti-CD19 chimeric antigen receptor. J Clin Oncol: Off J Am Soc Clin Oncol. (2015) 33:540–9. doi: 10.1200/JCO.2014.56.2025 PMC4322257 25154820

[135] Brentjens RJ Rivière I Park JH Davila ML Wang X Stefanski J. Safety and persistence of adoptively transferred autologous CD19-targeted T cells in patients with relapsed or chemotherapy refractory B-cell leukemias. Blood. (2011) 118:4817–28. doi: 10.1182/blood-2011-04-348540 PMC3208293 21849486

[136] Kochenderfer JN Dudley ME Carpenter RO Kassim SH Rose JJ Telford WG. Donor-derived CD19-targeted T cells cause regression of Malignancy persisting after allogeneic hematopoietic stem cell transplantation. Blood. (2013) 122:4129–39. doi: 10.1182/blood-2013-08-519413 PMC3862276 24055823

[137] Dai H Zhang W Li X Han Q Guo Y Zhang Y. Tolerance and efficacy of autologous or donor-derived T cells expressing CD19 chimeric antigen receptors in adult B-ALL with extramedullary leukemia. Oncoimmunology. (2015) 4:e1027469. doi: 10.1080/2162402X.2015.1027469 26451310 PMC4590028

[138] Morgan RA Yang JC Kitano M Dudley ME Laurencot CM Rosenberg SA. Case report of a serious adverse event following the administration of T cells transduced with a chimeric antigen receptor recognizing ERBB2. Mol Ther: J Am Soc Gene Ther. (2010) 18:843–51. doi: 10.1038/mt.2010.24 PMC2862534 20179677

[139] MacIver NJ Michalek RD Rathmell JC. Metabolic regulation of T lymphocytes. Annu Rev Immunol. (2013) 31:259–83. doi: 10.1146/annurev-immunol-032712-095956 PMC3606674 23298210

[140] Chang CH Qiu J O’Sullivan D Buck MD Noguchi T Curtis JD. Metabolic competition in the tumor microenvironment is a driver of cancer progression. Cell. (2015) 162:1229–41. doi: 10.1016/j.cell.2015.08.016 PMC4864363 26321679

[141] Angelin A Gil-de-Gómez L Dahiya S Jiao J Guo L Levine MH. Foxp3 reprograms T cell metabolism to function in low-glucose, high-lactate environments. Cell Metab. (2017) 25:1282–93.e7. doi: 10.1016/j.cmet.2016.12.018 28416194 PMC5462872

